# Local implementation of scalable malaria molecular surveillance in a low-resource setting: a North–South collaborative model

**DOI:** 10.1186/s12936-026-06040-6

**Published:** 2026-07-13

**Authors:** Martin Lukindu, Edward Lukyamuzi, Faisal Ashraf, Sanjay C. Nagi, Alfred Ssekagiri, Trevor Mugoya, Calvin Yee, Solome Mukwaya, Eric Lucas, Tony Nolan, Martin Donnelly, Jonathan Kayondo

**Affiliations:** 1 Department of Entomology, Uganda Virus Research Institute (UVRI), P.O.BOX 49, Entebbe, Uganda; 2 Department of Vector Biology, Liverpool School of Tropical Medicine, Liverpool, UK

## Abstract

Molecular surveillance of malaria vectors remains critically underutilized in sub-Saharan Africa, largely due to limited human capacity and technical resources, despite the region bearing the highest global burden of malaria. In a North–South collaborative effort, Uganda Virus Research Institute (UVRI) and Liverpool School of Tropical Medicine (LSTM) built capacity and improved on existing computational capabilities in line with molecular surveillance of *Anopheles gambiae*, the primary malaria vector in sub-Saharan Africa. We interrogated an amplicon panel capable of simultaneously detecting 55 Insecticide Resistance markers and 35 Ancestral Informative Markers, as well as polymorphisms in the *doublesex* gene—a key target for homing-based gene drives aimed at suppressing malaria vector populations. This represents a substantial advancement over conventional surveillance tools. The panel was tested and locally validated at UVRI with the lab colony and wild populations and a versatile data analysis pipeline for the panel was designed for raw data processing, analysis, and visualization. The tools and experience from this collaborative effort enhance molecular vector surveillance in the region and beyond, while also acting as a template for similar collaborations by other institutions.

## Introduction

Despite significant progress, malaria continues to plague Sub-Saharan Africa, accounting for over 90% of global cases and deaths [1] where transmission is sustained by a complex vectorial system of genetically diverse and highly adaptive mosquitoes of the genus Anopheles [2].

Current gains in malaria control have been driven by insecticide-based interventions including insecticide-treated nets and indoor residual spraying [3–7].

However, the widespread emergence of insecticide resistance poses a serious threat to the continued effectiveness of vector control interventions [8–11] and is partly blamed for the recent stagnation in malaria control in endemic countries. This challenge has necessitated the development of robust tools for managing insecticide resistance, particularly through the systematic monitoring of resistance-associated alleles and their spatial and temporal spread across endemic regions.

The stagnation in malaria control has also created a need for more innovative and complementary vector control interventions. tools that can reinvigorate the progress towards malaria elimination. Genetic control is fronted as potentially transformative addition to vector control, through two broad approaches aimed at spreading traits that affect the vector competence of malaria vectors through either population suppression or refractoriness to *Plasmodium* spp, the malaria causative pathogen.

Among these tools the trait is usually coupled to a gene drive designed to propagate into target populations [12–18]. Gene drives are selfish genetic elements that can bias their inheritance and rapidly invade a population. The most advanced types of gene drive rely on DNA endonucleases that are programmed (e.g., CRISPR) to copy themselves by recognizing and cutting target sequences within the mosquito genome.

Examining the genetic variation at the target sites both before and after deployment of these tools is essential in assessing their efficacy. Therefore, it makes sense to combine molecular surveillance tools that can build relevant combined capacity both immediately (for existing control tools) and in the future (for genetic control methods, such as gene drive). Hence there is an increasing need to strengthen molecular vector surveillance in malaria-endemic regions to monitor the emergence and spread of resistance, not only to existing interventions (e.g., indoor residual spraying and insecticide-treated nets) but also to emerging genetic vector control tools, such as genetic variations at target sites for gene drive-based mosquito control strategies.

The falling costs of massively parallel high-throughput DNA sequencing technologies [19–23] and the availability of computational capacity to store and analyze large genomic datasets (as reviewed [24]) have enabled the integration of genomics in malaria vector surveillance. Subsequently, vector genomics is emerging as an important field where genomic data is used to drive and inform vector control efforts. Malaria vector genomics holds great potential for various applications, including species identification, monitoring, and managing insecticide resistance, aiding novel genetic control strategies, studying adaptation to environmental and climatic changes, and conducting surveillance of vector populations, among others. Indeed, vector genomics is now part of a holistic malaria molecular surveillance strategy encompassing malaria vectors and the causative protozoa pathogens. Unfortunately, the potential of molecular vector surveillance in several institutions in sub-Saharan Africa is yet to be fully realized for several reasons including limited infrastructure and shortage of appropriately trained human capacity [25].

As a result of its historical focus on pathogen research, the ‘omics’ platforms and capacity at Uganda Virus Research Institute (UVRI) are more closely adapted to pathogen genomics than to disease vectors. Fortunately, this positions UVRI uniquely to broaden its ‘omics’ capacity by incorporating medically important vectors in Uganda. It is upon this background that the Malaria Molecular Surveillance (MMS) project was initiated as a collaborative endeavor between UVRI and Liverpool School of Tropical Medicine (LSTM), a UK-based center of excellence for research and education in tropical diseases. The overall project objective was to co-develop, validate, and establish at UVRI an integrated MMS platform for screening insecticide resistance and monitoring mosquito populations’ suitability for novel genetic control tools such as gene drives.

To achieve its overall objectives, the MMS project had two key activities: (i) Technology transfer between UVRI and LSTM that would entail training researchers and improvement of an amplicon sequencing data processing pipeline; (ii) Consolidation of internal genomics capacity, including adaptation of the UVRI genome center sequencing platforms for mosquito amplicon sequencing. We anticipate that this model would inspire similar north–south collaborative efforts to enhance ‘omics'-based research.

## Methods

### Context and existing capacities at UVRI

UVRI has a range of DNA sequencing capabilities from the conventional Sanger sequencing platform that caters to low coverage, short output reads to complex high-throughput next-generation, ‘short read’ sequencing and third generation long read’ sequencing platforms. Furthermore, UVRI has in place computational capacity for downstream genomic data processing, analysis, and storage. This has been possible partly through collaborative programs and/or projects. For example, UVRI’s Genomic Center—generously funded by the World Bank and the Defense Threat Reduction Agency (DTRA), with technical support from Los Alamos National Laboratory during its establishment—serves as a key resource. Also, through a collaboration with the UK’s Medical Research Council (MRC) and London School of Tropical Medicine and Hygiene (LSTMH) (https://www.mrcuganda.org/), UVRI launched the Uganda Genome Resource (UGR) which has enhanced Genome Wide Association Studies (GWAS) in Uganda albeit focusing on humans [26]. In addition, the Pan African Bioinformatics Network of the H3Africa Consortium, H3ABioNet [27], provided support for the acquisition, installation, and maintenance of High-Performance Computing (HPC) infrastructure while training UVRI staff in HPC operations and the development of analysis workflows. These efforts laid the foundation for robust next generation sequencing and bioinformatics capabilities, which were further strengthened through the eLwazi Open Data Science Platform (https://elwazi.org), ensuring continued infrastructure and bioinformatics support. These initiatives empowered UVRI to locally establish next generation sequencing, manage HPC systems and develop workflows for analyzing next-generation sequencing (NGS) data.

DNA sequencing platforms and computational systems at UVRI have mainly been used for research of viral pathogens, including Human Immunodeficiency Virus (HIV), Viral Hemorrhagic Fevers (VHFs), Arboviruses, and vaccine-preventable diseases such as Polio, Measles, and Rubella [28–36]. Here, the research focus is understanding virus evolution, surveillance of antiviral drug resistance, understanding virus transmission patterns within key populations, and discovery of novel viruses. Subsequently, the bioinformatics human capacity was also more aligned to manage and analyze the ever-growing volumes of pathogen-centric datasets generated by NGS-technologies so as to guide the Uganda public health policy. The MMS study leveraged this existing infrastructure and human capacity to incorporate vector genomics as part of the UVRI’S ‘omics’ capability specifically focusing on amplicon sequencing and its application in molecular vector surveillance.

### Capacity building activities

Genomic surveillance hinges on more than just advanced tools and technologies, it depends on equipping local researchers and institutions with the skills and resources to apply solutions effectively. Bridging gaps in technical expertise, infrastructure and data use is essential to empowering endemic regions to take charge in the fight against malaria. To strengthen the expertise required for effective genomic surveillance of malaria vectors in Uganda, we adopted a two-pronged capacity-building approach: hands-on wet lab training and remote computational collaboration. This strategy aimed to bridge existing skills gaps while promoting knowledge exchange and fostering a culture of collaborative innovation.

### In person training for wet lab training

With amplicon sequencing being a relatively new approach in malaria vector research, we organized an intensive 2-month training program hosted at LSTM’s Vector Biology Department. Facilitated by the Genomic Surveillance Unit (GSU) of the Wellcome Sanger Institute, the program brought together one staff from UVRI and LSTM to focus on the application of novel amplicon assays for profiling insecticide resistance in *Anopheles gambiae* populations. A novel amplicon panel, now designated as Ag-vampIR [37] was used in the amplification of targeted genomic regions *An*. *gambiae*.

**Fig. 1:**
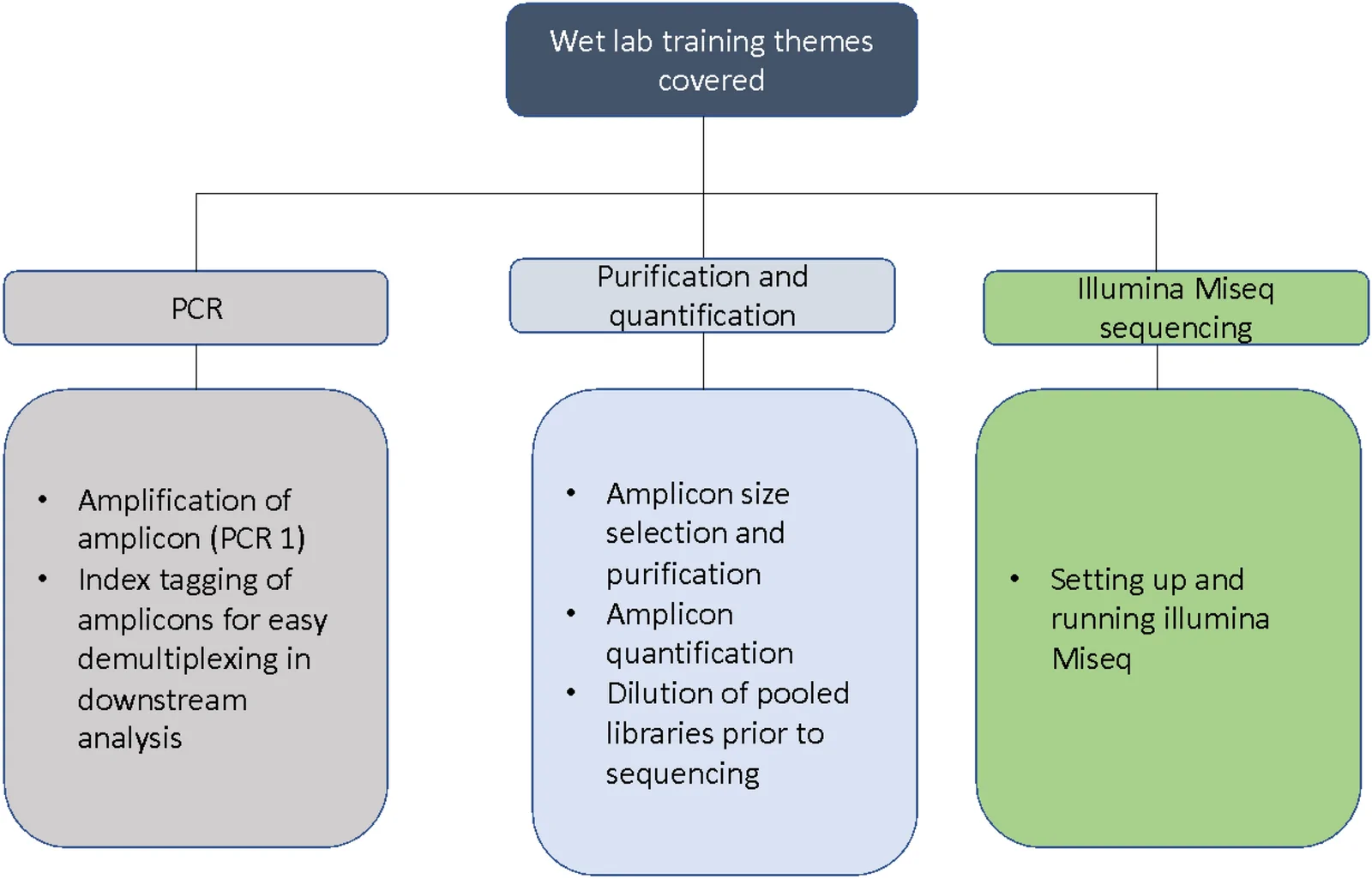
Themes and topics covered during human capacity training in amplicon sequencing

The training covered key components of NGS workflows, including DNA extraction, library preparation, sequencing, and quality control, as illustrated in Fig. 1. This hands-on approach provided participants with the technical confidence to independently execute these protocols while troubleshooting issues in real time.

### Co-development of UVRI’s computational capacity

While wet-lab training is vital for data generation, computational expertise is equally important for processing and analyzing the resulting genomic datasets, and as such, to complement the wet-lab training, we worked closely with LSTM to strengthen UVRI's computational infrastructure for vector genomic data processing. Recognizing the need to refine existing workflows, our collaboration was formalized through a series of goal-setting sessions aimed at aligning institutional objectives. Together, we developed a technology transfer plan that focused on adopting LSTM’s advanced vector genomic data processing workflows at UVRI.

**Fig. 2:**
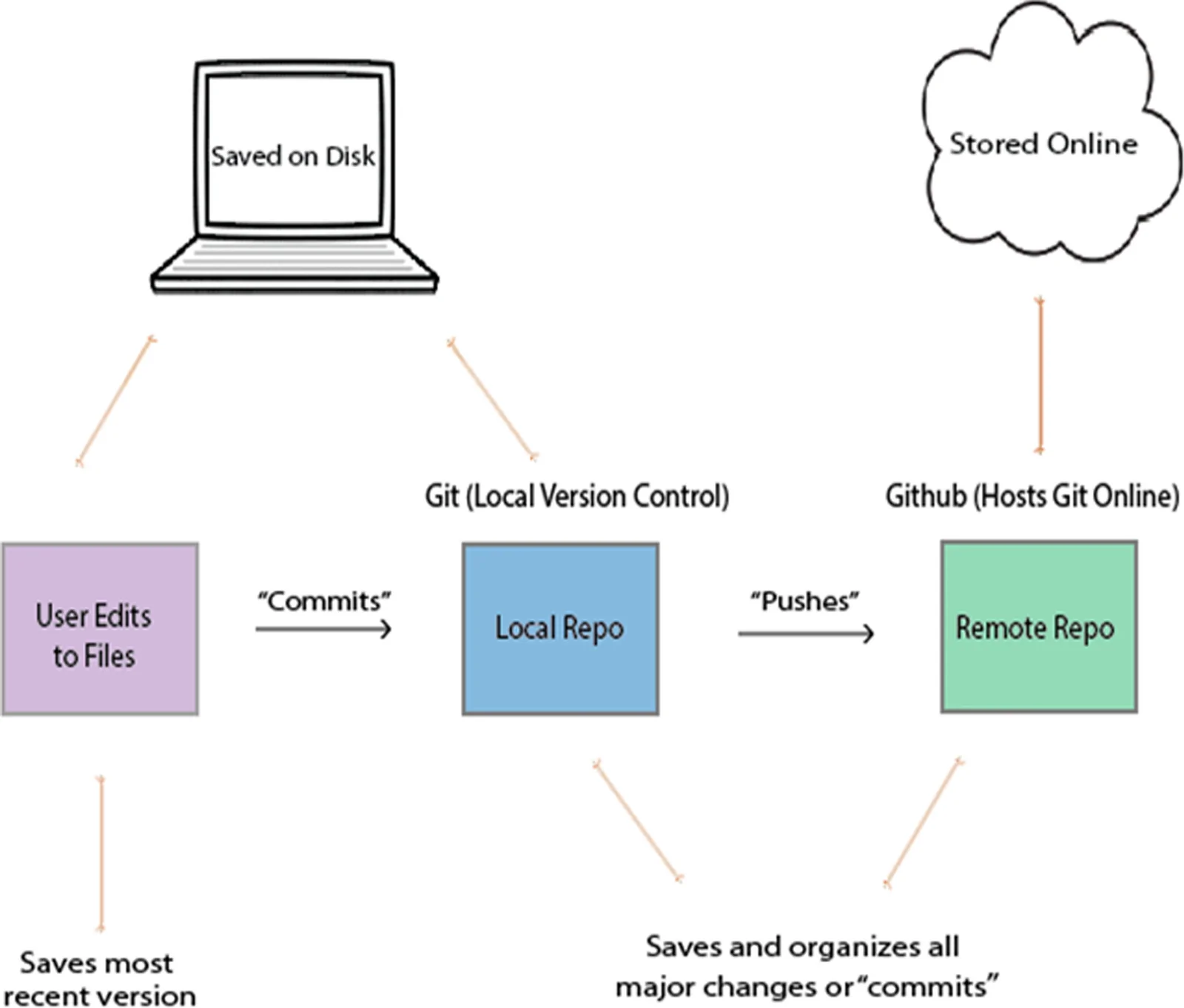
The collaborative framework using git facilitated open and transparent collaboration

Central to this plan was the use of collaborative frameworks, including Git [38] and GitHub [39] (as depicted in Fig. 2). These platforms have become indispensable for enabling remote collaboration models that support researchers in real-time collaborative tools and software development. Recent work highlights GitHub’s transformation from a software‑centric platform to a vital tool for real‑time, remote research collaboration, enabling integrated workflows spanning experiment planning, data analysis, code sharing, and manuscript development [40–44]. Leveraging such virtual platforms, bioinformatics experts are able to work closely with local teams without the constraints of geographical distance while ensuring continuous and transparent progress. GitHub provided a robust environment for seamless sharing of code, documentation, task management and allocation as well as updates between teams. The platform’s distributed version control system was particularly valuable in allowing structured collaboration during workflow development and optimization. Access control features ensured that project permissions were managed securely, safeguarding the integrity of the codebase while allowing team members appropriate access to contribute or review. Bug tracking and task management functionalities enabled the teams to log, prioritize, and address issues efficiently. These features facilitated streamlined communication and ensured that identified problems were systematically resolved. Additionally, the integration of continuous integration (CI) played a critical role in maintaining code quality. A CI pipeline automated the testing and validation of new updates, catching errors early and ensuring that changes did not disrupt the broader workflow.

### Design of the Ag-Vampir + dsx panel

We leveraged past published research for reference of IR markers [45] to interrogate and locally validate an amplicon SNP panel for monitoring insecticide resistance in *Anopheles gambiae* populations. The full set of 78 amplicon primers was designed and gifted by the Wellcome Sanger Institute [37]. We expanded the panel to also incorporate primers amplifying the intron 4-Exon 5 boundary of *Anopheles gambiae* double-sex gene (Agdsx) [46]. The intron 4-Exon 5 boundary of Agdsx is a potential target for gene drives designed to suppress of populations of *An*. *gambiae* [46]. Genetic variation at putative gene drive target sites within this locus may render gene drive constructs ineffective. Therefore, monitoring standing genetic variation or emergence of resistant variants, at the target sequence in the wild populations would be insightful for this novel genetic control approach.

Using in silico approaches, bioinformaticians at UVRI designed primers for amplification of this locus (intron 4-Exon 5 boundary). Primer design was performed in a Google Collaboratory environment using genomic data from the MalariaGEN *Anopheles gambiae* data resource (release 3.0). Design parameters, including amplicon size range, number of candidate primer pairs, and the target *Agdsx* locus, were defined as configurable notebook variables. Chromosome 2R sequence and variation data were loaded as custom xarray objects [47]. Primer design was conducted using AnoPrimer, a custom Python package that wraps Primer3 and incorporates allele frequency information at candidate primer binding sites. Primers were designed to flank the intron 4–exon 5 boundary and to encompass the T1–T3 gene drive target sites present in the best-performing population suppression gene drive to date [48]. Twenty candidate primer pairs were generated and evaluated using AG1000G variation data accessed via the MalariaGEN API (release 3.0) to assess alternate allele frequencies across Uganda, East Africa, and Africa. Primer pairs were further filtered based on amplicon size (150–210 bp), GC content, melting temperature compatibility, specificity via in silico PCR, and secondary structure analysis.

**Fig. 3:**
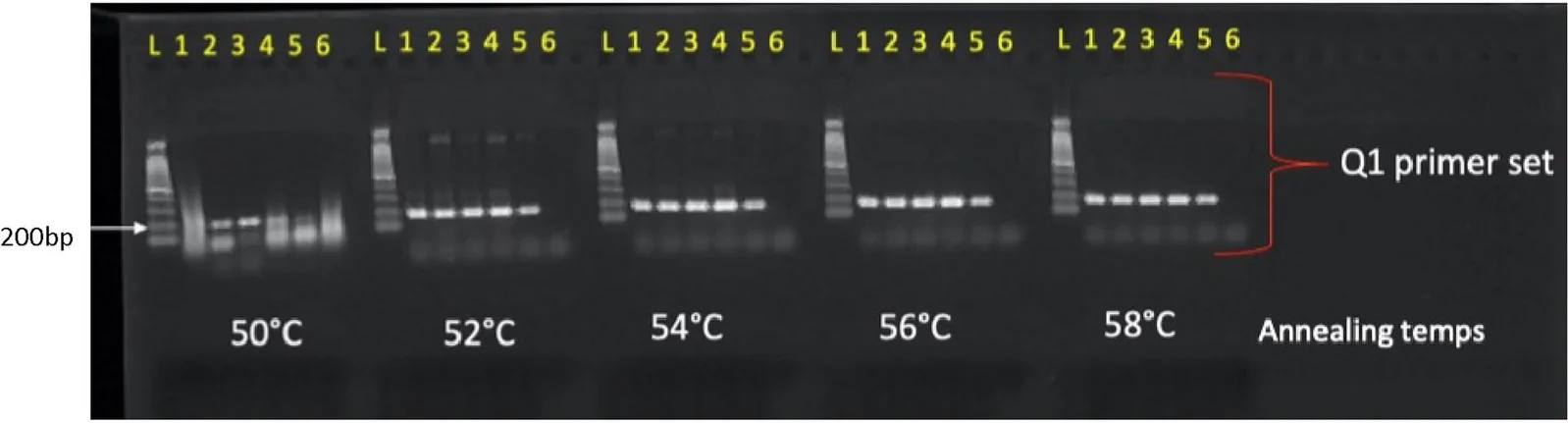
Agarose gel image showing successful PCR amplification of a 200 bp amplicon within the Agdsx intron 4-Exon 5 boundary at various annealing for one of the primers code named Q1. Lanes 1–5 show amplified *An. gambiae* amplicons; lane 6 represents the negative control, and L denotes the 100 bp DNA ladder

Ultimately, we selected two candidates for PCR amplification of the target sequence. Synthesis of the primers was done by Inqaba Biotec (Pretoria, South Africa) which were later optimized and validated using colonized *Anopheles gambiae* samples at UVRI and Fig. 3 shows the gel electrophoresis image of the amplicons.

### AmpSeeker pipeline co-development

**Fig. 4:**
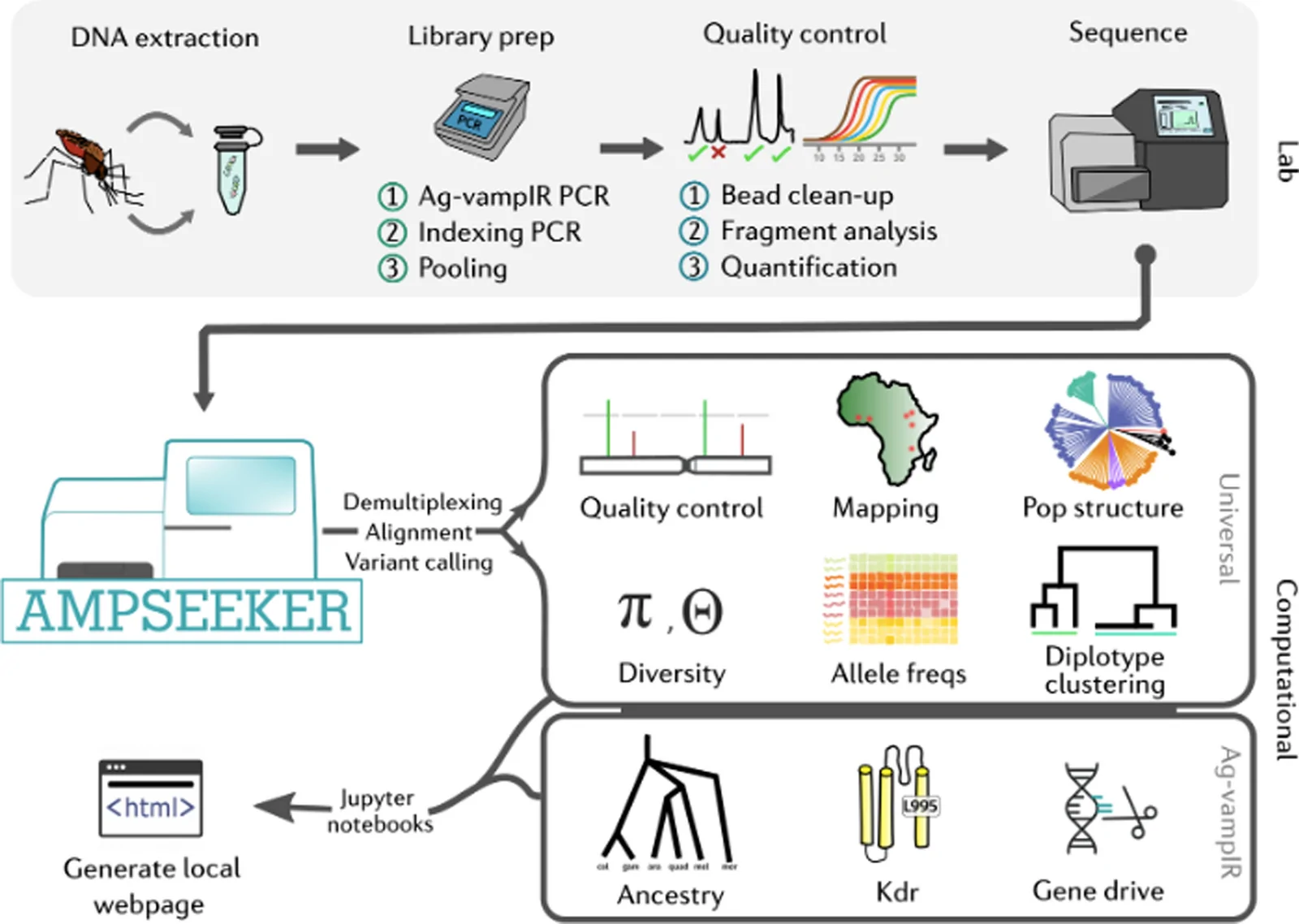
End-to-end molecular surveillance platform with AmpSeeker functionality (adapted from [37])

AmpSeeker, an open-source software (https://github.com/sanjaynagi/AmpSeeker) [37], was collaboratively designed by bioinformaticians at LSTM and UVRI as part of technology transfer and capacity building aspects of this project. During this collaborative effort, the AmpSeeker pipeline was refined to further enhance its applicability and utility in the context of mosquito genomics. The refinement process included the integration of various downstream analysis steps to facilitate genomics surveillance. One of the standout features of these enhancements is the ability to generate a local webpage with interactive plots and tables, providing a convenient platform for exploring results. This webpage, built using a combination of Papermill and Jupyter notebook [49], allows researchers and/or implementors to visualize key outputs, such as raw reads quality control metrics, alignment statistics, variant analyses, coverage data, interactive principal component analyses (PCAs), sampling site maps, allele frequency and genetic diversity plots (Fig. 4). These improvements make it easier to interpret complex genomic data outputs.

The schema, as depicted in Fig. 4, of the workflow highlights the expanded capabilities of AmpSeeker, which includes a wider array of analytical processes. These include genome alignment, variant calling, variant quality control, coverage statistics, and visualization in IGV (Integrative Genomics Viewer). Also, the pipeline supports advanced analyses such as allele frequency calculation, genetic diversity assessments, Kdr origin analysis, and species assignment.

Testing and validation of the AmpSeeker pipeline was done using genomic datasests of *Anopheles gambiae* in publicly available datasets including ag1000 and ANOSPP project at the Wellcome Sanger Institute [50]. The newly refined AmpSeeker pipeline was applied in a local context at UVRI to analyze local mosquito samples (see below).

## Results

### Human capacity development

One staff member at UVRI gained hands-on experience in wet-lab methodologies and spearheaded the replication of the amplicon assays at UVRI, demonstrating technology transfer between the two institutions. In addition, three UVRI staff members collaborated with colleagues at LSTM to co-develop and validate the AmpSeeker bioinformatic, contributing to its robustness and cross-institutional reproducibility.

### Local implementation of Ag-vampIR panel and AmpSeeker analysis

We successfully replicated the wet-laboratory procedures previously conducted at LSTM during the amplicon sequencing training. A total of 80 amplicon targets (of the Ag-vampIR panel) were amplified from genomic DNA extracted from a set of *Anopheles gambiae* specimens collected in Uganda and sequenced using an Illumina MiSeq Nano v2 flow cell (2 × 150 bp).

As part of adapting the sequencing platform at UVRI to vector genomics, we tagged each mosquito sample and 96 well-plate with unique i5 and i7 indices. Tagging enabled pooling of samples in a single flow cell during Illumina sequencing. It also facilitated sequence demultiplexing during downstream analysis. Sequencing was performed on an Illumina Miseq platform following manufacturer’s instructions.

Sequencing quality was moderately high, with slightly over 80% of bases achieving Q30 and 95.9% of reads passing quality filters. The sequencing run yielded a total output of 310 Mb, within the Illumina specification of 300 Mb, and generated 2.02 million FASTQ reads, consistent with expectations for a MiSeq Nano v2 flow cell (~ 2 million reads).

**Fig. 5:**
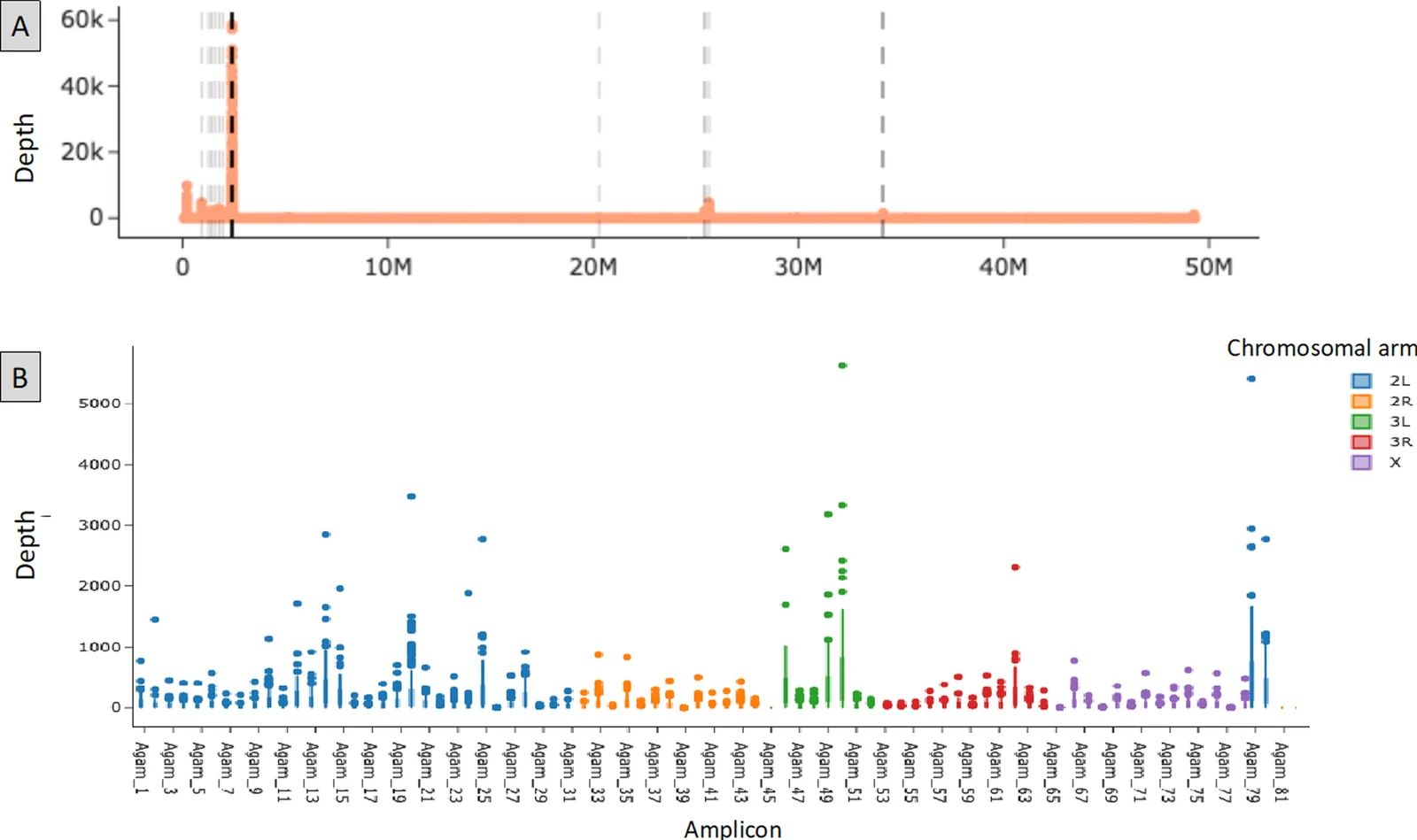
Plots for a validation run conducted at UVRI; **A** Target region specificity for amplicons at the left arm of chromosome 2; **B** Coverage at target amplicons at chromosomal arms of *Anopheles gambiae*

Initial analysis of this dataset using the AmpSeeker pipeline demonstrated that the majority of amplicons were successfully amplified at their respective target regions (e.g. Fig. 5A), with coverage observed across most target chromosomal arms and at relatively high sequencing depth (Fig. 5B).

Although the potential of the Ag-vampIR panel and the AmpSeeker pipeline has also been reported in Nagi et al. [37], we anticipate further scaling up of this study by incorporating additional datasets from malaria-endemic regions in Uganda and by implementing the full suite of AmpSeeker pipeline features. These will include principal component analysis (PCA), allele-frequency estimation, and assessments of genetic diversity, among others, which are beyond the scope of this initial report.

## Discussion

### Successes and challenges

The partnership between UVRI and the LSTM has been transformative in advancing genomic surveillance capacity at UVRI, fostering significant milestones in technical capacity development and practical applications for malaria vector surveillance. A major success was the improvement of UVRI's expertise in amplicon sequencing and genomic data processing. The hands-on training program conducted at LSTM equipped UVRI staff member with a solid foundation in amplicon sequencing techniques, who in turn trained additional local colleagues, enabling them to generate and interpret data critical for monitoring insecticide resistance in Anopheles populations.

Cascading the knowledge to additional local staff catalyzed broader capacity-building in vector genomics. Complementary remote collaborations further strengthened technical capabilities, with UVRI contributing to developing an advanced data processing workflows through an open, collaborative frameworks.

Beyond technical achievements, the collaboration underscored the importance of fostering a culture of open communication and mutual learning. Regular goal-setting sessions and transparent sharing of resources allowed all partners to contribute to and benefit from the initiative, setting the stage for long-term success in vector genomic surveillance efforts.

### Lessons learned and future prospects

Our collaborative journey toward advancing molecular surveillance at UVRI has provided invaluable insights into practical strategies for knowledge transfer and the establishment of sustainable platforms in resource-limited settings. These lessons serve as a guide for similar efforts in other malaria endemic regions, where capacity building and infrastructure development are critical to combating malaria. A key takeaway from the collaboration is the importance of tailored, hands-on training programs in driving meaningful skill acquisition.

The structured use of virtual platforms also ensured that knowledge transfer extended beyond isolated workshops, evolving into an ongoing, dynamic process.

As with all initiatives, there’s a critical challenge that requires addressing—how do we build resilient platforms equipped to address the evolving challenges of malaria control? Sustainability in molecular surveillance requires more than technical expertise; it demands a systems-level approach that addresses infrastructure, human capacity, and strategic alignment. Investments in scalable data storage and computational infrastructure emerge as a critical component to enable institutions in Low- and Middle-Income Countries to manage and analyze large volumes of genomic data efficiently. Cloud-based solutions and partnerships with established institutions offered a practical pathway to address immediate resource gaps while planning long-term infrastructure development.

### Prospects and NMCP integration

As we look ahead, the journey to fully integrate malaria molecular surveillance into public health programs and amplify its impact through enhanced collaborations presents both opportunities and challenges. These next steps are essential to ensure that the gains made in genomic capacity and technology transfer translate into meaningful outcomes for malaria control and elimination efforts. The integration of malaria molecular surveillance into public health workflows remains an ongoing effort. A critical step is fostering deeper engagement with National Malaria Control Programs (NMCPs) to align genomic insights with actionable policy decisions. This requires streamlining data processing to deliver timely and user-friendly outputs that address specific public health priorities such as insecticide resistance management and intervention efficacy monitoring.

On the policy front, continued engagement with regional and international malaria stakeholders can elevate the importance of molecular surveillance on the global health agenda. Collaborative workshops, publications, and case studies demonstrating the impact of genomic data on vector control outcomes can drive awareness and foster policy alignment. The future of molecular surveillance lies in its potential to transform malaria control into a more precise and adaptive endeavor.

## Conclusion

Our work underscores the value of the North–South collaboration model in building sustainable genomics capacity for malaria vector surveillance. By integrating technical expertise, infrastructure development, and targeted training, we established a framework that not only addressed immediate skill gaps but also cultivated long-term knowledge exchange. This collaborative approach empowered our local team to take ownership of genomic surveillance efforts with the intent of reducing dependency on external support and foster independent research and data analysis.

A key achievement of this partnership was the development and successful deployment of bioinformatics tools tailored for molecular surveillance. Genomic workflows were developed and optimized to better fit operational realities to improve the efficiency, scalability and accuracy of vector population monitoring. These refined tools enable the tracking of key genetic markers linked to insecticide resistance, population structure, and vector species identification which provide critical insights for guiding targeted vector control interventions. The possibility of integrating these genomic approaches into routine surveillance programs highlights the transformative role of genomics in malaria control, demonstrating its potential to offer real-time, data-driven guidance for intervention strategies. Ultimately, this initiative demonstrated that investing in regional capacity through training, infrastructure support and collaborative knowledge-sharing can accelerate the adoption of genomics-based surveillance in malaria-endemic regions. Moving forward, sustained partnerships and strategic investments will be essential to ensure that these advancements continue to drive impactful, locally-led malaria control efforts.

## Data Availability

No datasets were generated or analysed during the current study.
